# The Interaction between *Nidovirales* and Autophagy Components

**DOI:** 10.3390/v9070182

**Published:** 2017-07-11

**Authors:** Yingying Cong, Pauline Verlhac, Fulvio Reggiori

**Affiliations:** Department of Cell Biology, University of Groningen, University Medical Center Groningen, A. Deusinglaan 1, 9713 AV Groningen, The Netherlands; y.cong@umcg.nl (Y.C.); p.a.verlhac@umcg.nl (P.V.)

**Keywords:** coronavirus, arterivirus, mesonivirus, ronivirus, autophagosome, autophagic flux, infection, replication, egression

## Abstract

Autophagy is a conserved intracellular catabolic pathway that allows cells to maintain homeostasis through the degradation of deleterious components via specialized double-membrane vesicles called autophagosomes. During the past decades, it has been revealed that numerous pathogens, including viruses, usurp autophagy in order to promote their propagation. *Nidovirales* are an order of enveloped viruses with large single-stranded positive RNA genomes. Four virus families (*Arterividae*, *Coronaviridae*, *Mesoniviridae,* and *Roniviridae*) are part of this order, which comprises several human and animal pathogens of medical and veterinary importance. In host cells, *Nidovirales* induce membrane rearrangements including autophagosome formation. The relevance and putative mechanism of autophagy usurpation, however, remain largely elusive. Here, we review the current knowledge about the possible interplay between *Nidovirales* and autophagy.

## 1. The Order of *Nidovirales*

**Figure 1:**
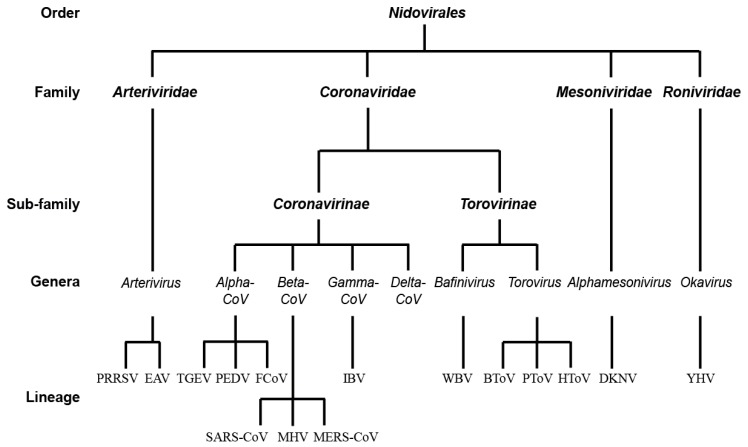
The taxonomy of the order *Nidovirales*. BToV, bovine torovirus; DKNV, dak nong virus; EAV, equine arteritis virus; FCoV, feline coronavirus; HToV, human torovirus; IBV, infectious bronchitis virus; MERS-CoV, Middle East respiratory syndrome coronavirus; MHV, mouse hepatitis virus; PRRSV, porcine reproductive and respiratory syndrome virus; PEDV, porcine epidemic diarrhea virus; PToV, procine torovirus; TGEV, transmissible gastroenteritis coronavirus; SARS-CoV, severe acute respiratory syndrome coronavirus; YHV, yellow head virus; WBV, white bream virus.

*Nidovirales* is an order of enveloped, single-stranded positive genomic RNA viruses. They have the largest known viral RNA genomes and infect a broad range of hosts [1]. The order of *Nidovirales* includes four virus families: *Roniviridae*, *Arterividae*, *Mesoniviridae*, and *Coronaviridae* (Figure 1). This classification is principally based on the organization of their viral genome, the closeness in genome sequences, the antigenic properties of the viral proteins, the replication strategy, the structure and physicochemical properties of the virions, the natural host range, the cell and tissue tropism, the pathogenicity, the cytopathology, and the mode of transmission [1,2,3,4]. The name of *Nidovirales*, from the Latin word “nidus” for nest, refers to a nested set of viral subgenomic messenger RNAs that is produced during infection [5]. Within the *Coronaviridae* family, the subfamily *Coronavirinae* is the one encompassing the larger number of viruses. Species in this subfamily, which include several human pathogens, can be grouped into four main subgroups on the basis of serological and genetic properties: *Alphacoronavirus*, *Betacoronavirus*, *Gammacoronavirus*, and *Deltacoronavirus* [6,7,8] (Figure 1). *Torovirinae* is also a subfamily of *Coronaviridae* and four *Torovirus* species have been identified so far: the equine, bovine, porcine, and human *Toroviruses* [9,10] (Figure 1). The *Mesoniviridae* subfamily has one genus, which contains one species, the *Alphamesonivirus*. *Alphamesonivirus* are mosquito-specific viruses with extensive geographic distribution and host range [11]. Their virions are 60–80 nm in diameter, with club-shaped surface spikes and consist of eight major structural proteins, including a nucleocapsid protein, four differentially glycosylated forms of the membrane protein, and the spike S protein [12,13]. *Roniviridae* contain the genus *Okavirus* and although still little is known about them, the yellow head virus (YHV) can cause significant economic losses to the shrimp industry and is listed as a notifiable disease by the World Organization for Animal Health [1,14]. In recent years, veterinarians have also become very concerned about *Arterividae*, in particular the porcine reproductive and respiratory syndrome virus (PRRSV), which is causing economic losses to the USA swine industry that are estimated to US$560 million per year [15,16].

**Figure 2:**
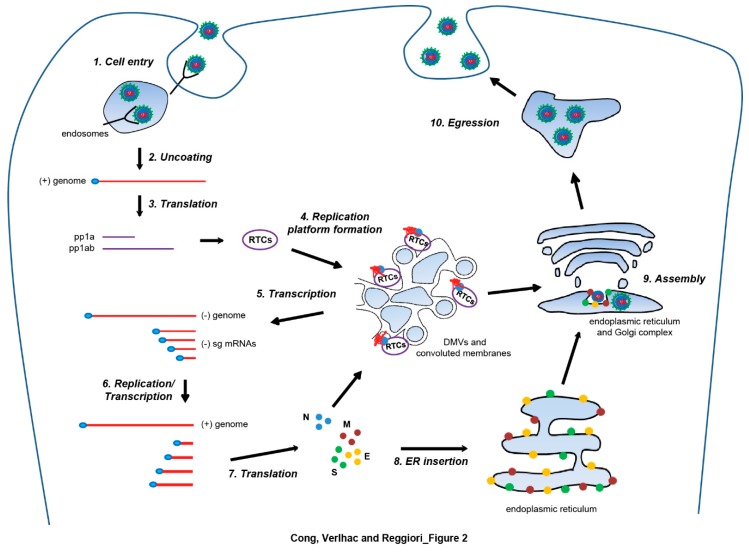
Generalization of *Nidovirales* life cycle, based on the information acquired studying *Arteriviruses* and *Coronaviruses*. Infection starts with the binding of the viral particle to a cell surface receptor and subsequent cell entry through membrane fusion in endosomes upon endocytosis (step 1). Translation of the released genomic RNA (gRNA) yields replicase polyproteins (step 2), i.e., polyprotein 1a (pp1a) and polyprotein 1ab (pp1ab), which undergo autoproteolytic processing to generate nonstructural proteins that assemble into replication-transcription complexes (RTCs). The RTCs are part of a complex membranous network composed of double membrane vesicles (DMVs) and convoluted membranes (step 4). The RTCs first engage in minus-strand RNA synthesis to produce both single strand full-length and subgenomic (sg) minus-strand RNAs (step 5). Subsequently, they use sg mRNAs as templates for the production of the gRNA and plus-strand sg mRNAs required to express the structural protein genes (step 6). Newly synthesized S, E, and M structural proteins are inserted in the endoplasmic reticulum (ER) (steps 7 and 8), whereas the N nucleocapsides are translated and oligomerize in the cytosol, where they interact with RTCs and associate with the gRNA to form the ribonucleoprotein complexes (step 7). Virion assembly takes place in the ER and/or Golgi (step 9), and involves the inward budding of the limiting membrane of these compartments, which is triggered by the interaction between the structural proteins and the ribonucleoprotein complexes. Mature virions are released extracellularly by exocytosis (step 10).

*Nidoviruses* rank among the most complex RNA viruses and their molecular genetics clearly discriminates them from other RNA virus orders [1]. Still, our knowledge about their life cycle, mostly unveiled with studies on *Coronaviruses* (*CoVs*), is very limited [1,3,17,18]. To enter cells, *Nidoviruses* bind to cell surface receptors, an event that precedes the fusion of the viral and cellular membranes (Figure 2, step 1), which is presumably mediated by one of the surface glycoproteins [19,20]. The fusion takes place either at the plasma membrane or in the endosomes and releases the nucleocapsid into the host cell cytoplasm [19] (Figure 2, step 1). After genomic RNA uncoating from the nucleocapsid, two large replicase open reading frames (ORFs), ORF1a and ORF1b, are translated by host ribosomes to yield two large polyprotein precursors that undergo autoproteolytic processing to eventually produce the non-structural (nsp) proteins. The nsp proteins interfere with the host defenses but also induce the formation of double-membrane vesicles (DMVs) and convoluted membranes, on which they collectively form the replication-transcription complexes (RTCs) [19,20] (Figure 2, steps 2, 3, and 4). These complexes mediate the synthesis of the genomic RNA and a nested set of subgenomic RNAs that directs the translation of the structural proteins (the nucleocapsid N protein, the membrane M protein, the envelope E protein and the spike S protein) and some accessory proteins, like the hemagglutinin esterase in the case of Severe Acute Respiratory Syndrome (SARS)-CoV or Mouse Hepatitis Virus (MHV) [21,22,23] (Figure 2, steps 5 and 6). Newly synthesized genomic RNAs associate with the cytoplasmic nucleocapsid proteins to generate the so-called ribonucleoprotein complexes [20,22]. The viral structural envelope proteins are inserted into endoplasmic reticulum (ER) and targeted to the site of virus assembly, the ER, or the Golgi, where they interact with the ribonucleoprotein complex to initiate the budding of virus particles into the lumen of the membrane compartment [20,24,25] (Figure 2, steps 7, 8 and 9). Newly formed virions then egress the host cell through secretion via the exocytic pathway [20,24] (Figure 2, step 10).

## 2. Autophagy and the Autophagy-Related Proteins

**Figure 3:**
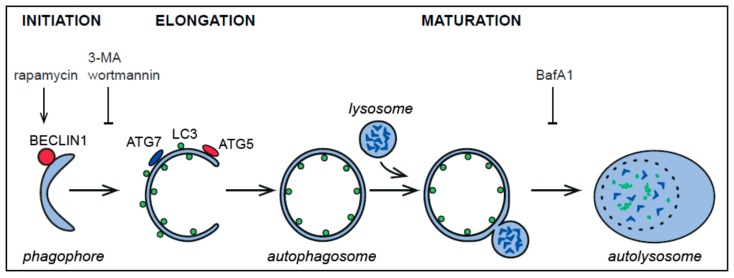
Overview of autophagy in mammalian cells. Schematic representation of the autophagic flux, some of its main regulators and of the effects of compounds commonly used to modulate autophagy. 3-MA: 3-methyladenine; BafA_1_: bafilomycin A_1_, LC3: microtubule-associated protein light chain 3.

Within the term autophagy are grouped all those catabolic pathways mediating the delivery of cytoplasmic material into the mammalian or plant/yeast vacuole for degradation. There are three major types of autophagy, i.e., macroautophagy, microautophagy, and chaperone-mediated autophagy [26]. Macroautophagy (hereafter referred to as autophagy) is conserved among eukaryotes that allows the turnover of excess or damaged cellular components, including long-lived proteins and organelles, to maintain cellular energy levels and ensure survival [27,28,29,30,31,32,33,34,35]. This process is characterized by double-membrane vesicles called autophagosomes, which sequester the cytoplasmic components targeted to destruction and deliver them into lysosomes for degradation [33,34,35,36,37] (Figure 3). The process can be divided into three different steps: The initiation step, when the phagophore or isolation membrane is formed, the elongation step, during which the phagophore expands and close to generate an autophagosome, and the maturation step, where the autophagosome fuses with the lysosome (Figure 3). Autophagy is regulated by several signaling cascades, including the one centered on the mammalian target of rapamycin (mTOR) [34,35,38]. Autophagosomes are formed through the concerted action of the autophagy-related (*ATG*) genes [29,33]. The proteins encoded by the *ATG* genes have been classified in five functional groups. The unc-51 like autophagy activating kinase (ULK) kinase complex, the class III hVPS34 phosphatidylinositol 3-kinase complex, and the ATG9 cycling system, on one hand, play a key role in the initiation of autophagy and phagophore formation. The ATG12 and microtubule-associated protein light chain 3 (LC3) conjugation systems, on the other hand, mediate the elongation and closure of the phagophore. The first of these ATG protein complexes responds to upstream regulatory signals, such as the inactivation of mTOR, and key in initiating the formation of an autophagosome, is the ULK kinase complex, which is composed of ULK1 or ULK2, ATG13, FAK family kinase-interacting protein of 200 kDa (FIP200), and ATG101 [31,39]. The class III hVPS34 phosphatidylinositol 3-kinase, which is part of a complex including hVPS15, ATG14L1, and BECLIN1, generates the pool of phosphatidylinositol 3-phosphate (PtdIns3P) on autophagosomal membranes that facilitates the recruitment of PtdIns3P effectors such as double FYVE-containing protein 1 (DFCP1) and the human WD-repeat protein interacting with phosphoinositides (WIPI) proteins [40,41,42,43,44,45]. The hVPS34-containing complex as well as the transmembrane protein ATG9, are two other important factors during the early stage of autophagosome biogenesis [46,47]. Subsequently, two ubiquitination-like systems, which ultimately recruit members of the LC3/ATG8 protein family onto autophagosomal membranes through their conjugation to phosphatidylethanolamine, are essential for the completion of the forming autophagosomes [47]. Finally, the fusion of autophagosomes first with endosomes and then with lysosomes, leads to the formation of amphisomes and autolysosomes, respectively, where the degradation of the autophagy cargoes take place [37].

It has long been believed that the ATG proteome is exclusively involved in autophagy, but recent findings have revealed that single *ATG* genes or functional clusters of *ATG* genes fulfill important cellular functions outside the context of their role in autophagy [48,49,50]. Some of these functions have been discovered by studying host–pathogen interactions [48,49,51]. For example, ATG5 but no other ATG proteins play a unique role in the defense against *Mycobacterium* infection [52]. More recently, it has been shown that FIP200 and ATG13 participate in the controlling of picornaviral infections outside the context of autophagy [53].

## 3. *Nidovirales* and Autophagy

Here, we will review the current knowledge on the interplay between *Nidovirales* and autophagy. There are currently no data available for several viruses and few *Nidovirales* families. Thus, this compendium will focus on the documented viruses in the *Arterivirus* and *Coronavirus* families (summarized in Table 1).

### 3.1. Arteriviruses and Autophagy

The two most studied *Arteriviridae* are PRRSV and the equine arteritis virus (EAV). The PRRSV strain, which was historically first characterized and is commonly referred to as atypical (i.e., AP PRRSV), causes the abortions in 10–50% of the sows, and fever and anorexia leading to the death of 5–10% of them [69]. However, in 2006, the emergence of a novel virulent highly pathogenic PRRSV (HP PRRSV) strain, carrying mutations in *nsp1β*, *nsp2*, and *ORF5* genes, caused higher morbidity (50%) and mortality (20%) rates in piglets and sows [70]. The equine arteritis virus, in contrast, infects horses and donkeys, and can cause abortions in pregnant females and mortality in neonates [71].

The first study on the role of autophagy in *Arterivirus* life cycle was carried out with the HP PRRSV strain [57]. Infected cells displayed the presence of a higher number of autophagosome-like double-membrane vesicles, an accumulation of green fluorescent protein (GFP)-LC3-positive puncta and higher levels of lipidated LC3, indicating an induction of autophagy [57]. Inhibition of autophagy with either 3-methyladenine (3-MA), a non-specific hVPS34 inhibitor, or depletion of *ATG7* or *BECLIN1*, led to a significant reduction in both expression of PRRSV *nsp2* and PRRSV titer. Conversely, induction of bulk autophagy using rapamycin (mTOR inhibitor) resulted in an enhancement of viral replication [57], a result that later was confirmed by others [57,58,59,60]. In one of these subsequent studies, Pujhari et al. showed that while the virus titer in the supernatants of infected cells treated with rapamycin was higher than in the control, intracellular levels of PRRSV N protein or nsp2 assessed by flow cytometry were lower [58]. This latter result is the opposite of the ones obtained by the other studies where rapamycin treatment led to an up-regulation of *nsp2* expression [57,58,59,60].

Two subsequent works reached the same conclusion of autophagy having a beneficial role in PRRSV life cycle [59,60]. Liu et al. confirmed autophagy induction by both AP and HP PRRSV strains [59]. Interestingly, they also observed a decrease in the virus titer in cells treated with bafilomycin A_1_ (BafA1), a drug inhibiting either autophagosome-lysosome fusion or lysosomal degradation, which suggested that autolysosomes might serve as replication platforms for PRRSV [59]. In contrast, Sun et al. showed, using confocal microscopy, that the HP PRRSV infection inhibits the fusion between autophagosomes and lysosomes [60]. This result indicates that PRRSV might trigger an incomplete autophagy and implicates that this could be beneficial for the virus life cycle. To gain insights into a possible molecular connection between autophagy and PRRSV, they also transfected cells with either *nsp2* or *nsp3*, which encode two transmembrane components of *Arterivirus* RTCs that play a central role in DMVs formation [63]. Interestingly, they found the co-localization between endogenous LC3 and ectopically expressed nsp2, but not nsp3, by confocal microscopy and fractionation on continuous density gradients, suggesting that the accumulated autophagosomes during PRRSV could represent the replicative platforms [60]. Thus, it still remains to be firmly demonstrated whether the results obtained with the ectopic expression of *nsp2* recapitulates a situation occurring in cells exposed to PRRSV.

Recently, three additional research articles provided evidences that autophagy but also apoptosis are induced by PRRSV in host cells [61,62,64]. Wang and collaborators investigated apoptosis and autophagy activation in the thymus of piglets infected with the HP PRRSV strain because they previously showed that this virus leads to thymic atrophy and thymocyte apoptosis [61]. Their investigation concluded that the HP PRRSV could induce apoptosis in bystander cells and autophagy in both infected and bystander cells in the thymus of infected piglets [61]. In a follow-up study, another laboratory found that HP PRRSV replication was attenuated in autophagy deficient Marc-145 cells and potentiated by inhibiting apoptosis using z-VAD-fmk, a caspase pan-inhibitor [62]. Interestingly, HP PRRSV replication could be restored in the autophagy deficient cells by blocking apoptosis, suggesting a functional interplay between autophagy and apoptosis during PRRSV replication. Subsequently, Zhou et al. confirmed the activation of autophagy and a subsequent induction of apoptosis over the course of a PRRSV infection in Marc-145 cells [64]. In their study, inhibition of autophagy by 3-MA caused a significant increase in PRRSV-induced apoptosis, also unveiling a potential connection between both mechanisms. In line with this conclusion, they also observed an increase in the expression of Bcl-2-associated death promoter (BAD), a pro-apoptosis protein, and BECLIN1, an autophagy regulator. Interestingly, co-immunoprecipitation and confocal microscopy experiments revealed the formation of a BAD-BECLIN1 complex in infected cells [64]. *BECLIN1* knockdown significantly decreased viral replication and PRRSV-induced autophagy as expected, while knocking down of BAD resulted in an induction of autophagy and enhanced viral replication [64]. The authors concluded that the enhancement of autophagy could promote PRRSV replication by postponing apoptosis through the formation of the BAD-BECLIN1 complex [64].

In a study exploring a potential connection between EAV and autophagy, Monastyrska et al. found that the DMVs induced by this virus are decorated with LC3 but the EAV life cycle proceeded unaffected in cells lacking *ATG7* [68]. Although autophagy was not required, depletion of LC3 markedly reduced EAV replication and it could be fully restored by expression of a non-lipidable form of LC3 [68]. Similar to MHV, EAV-induced DMVs were also positive for EDEM1 (ER Degradation Enhancing Alpha-Mannosidase Like Protein 1) leading to the conclusion that EAV might also hijack the ER-derived membranes of EDEMosomes to ensure its replication [67,68]. It still needs to be investigated, however, whether other ATG proteins are dispensable for EAV life cycle. Furthermore, it is unclear whether both autophagy and apoptosis are induced over the course of an EAV infection as observed for PRRSV.

### 3.2. Alphacoronaviruses and Autophagy

The most studied *alphacoronaviruses* (*alpha-CoVs)* are the Transmissible Gastroenteritis Virus (TGEV) and Porcine Epidemic Diarrhea Virus (PEDV), which both infect suckling piglets and lead to a high mortality rate [72,73]. Recurrent outbreaks of PEDV have occurred across Asia and the USA, causing significant economic losses [73]. *Alpha-CoV* also includes human pathogens such as HCoV-229E and HCoV-NL63, which are associated with respiratory tract infections such as the common cold to bronchiolitis [74,75]. Despite their medical and veterinary relevance, however, the exact mechanisms of *alpha-CoV* replication and pathogenesis are not well characterized yet.

Sun et al. recently performed a high throughput mass spectrometry analysis in PEDV-infected Vero cells [76]. Their goal was to identify which cellular proteins are differentially expressed during viral infection to better understand the impact of PEDV on host cells. Interestingly they found that autophagy might be among the altered pathways as sequestrosome 1 (SQTSM1/p62) and LC3 expression levels were upregulated. A subsequent study thus focused on the potential interplay between autophagy and PEDV [77]. The authors found that PEDV infection induces autophagy in Vero cells using different assays such as LC3-positive puncta formation, transmission electron microscopy (TEM) and western blot assessment of both LC3 lipidation and SQSTM1/p62 turnover. In line with these observations, 3-MA treatment or the ablation of either *BECLIN1* or *ATG7*, reduced the production of infectious viral particles. Treatment of the infected cells with rapamycin, however, did not change the viral titer, probably because of the multiple effects of this compound on the cell physiology. Altogether, these data showed that autophagy induction during PEDV infection could be beneficial for the virus.

Two more recent studies have addressed the link between autophagy and TGEV replication but they have reached opposite conclusions [78,79]. They both demonstrated that autophagy is induced in TGEV-infected cells using methods such as TEM, LC3 puncta formation and western blot analysis of both LC3 lipidation and SQSTM1/p62 degradation. In their article Zhu et al. also showed that the selective degradation of mitochondria by autophagy, i.e. mitophagy, might be induced by TGEV as they observed in infected IPEC-J2 cells a reduced mitochondrial mass, a light oxidative stress, and mitochondria in autophagosome-like vesicles [79]. In support of this notion, the authors also found that TGEV N protein and GFP-LC3 localize to mitochondria. Interestingly, induction of mitophagy by mitochondria depolarization using carbonyl cyanide m-chlorophenyl hydrazone (CCCP) increased the viral titer, suggesting that this pathway might be beneficial for viral replication. Similarly, induction of bulk autophagy using rapamycin also led to more production of progeny virus [79]. Conversely, incubation with 3-MA or *ATG5* depletion inhibited viral replication assessed by N protein expression and viral titers. Zhu et al. thus concluded that autophagy, and mitophagy in particular, plays an important role in TGEV life cycle [79]. This conclusion, however, differs from the one reached in a parallel study. Guo and collaborators found that both hVPS34 and lysosomal inhibitors increased both the number of cells infected by TGEV and the viral titer, while rapamycin had an opposite effect [78]. Moreover, silencing *LC3*, *ATG5,* or *ATG7* expression in ST cells promoted TGEV replication, showing that autophagy has an antiviral function [78]. The apparent discrepancies between these two studies could be explained by the use of different TGEV strains (SHXB versus H165) and/or cell lines (IPEC-J2 versus ST). Further investigations are thus needed to conclusively determine whether autophagy plays a role in TGEV life cycle. It will be particularly important to establish this in tissues that are normally infected by PEDV.

### 3.3. Betacoronaviruses and Autophagy

The first investigations on the interplay between *CoV* and autophagy focused on MHV, a *betacoronavirus* (*beta-CoV*) that is often used as a model virus to study the mechanism of *CoV* infections. As a result, there is a relatively large amount of data about various aspects of MHV life cycle. Importantly, the genus *beta-CoV* also includes the highly pathogenic human viruses SARS-CoV and MERS-CoV, two viruses that cause acute respiratory symptoms and they are on the WHO list of viruses likely to cause future epidemics (Figure 1).

Like other *CoV*, MHV replication takes place on interconnected structures formed by convoluted membranes and DMVs, with the latter being reminiscent of autophagosomes [80]. This structural similarity prompted the investigation of a possible interplay between autophagy and *CoV* replication. Interestingly, the first two studies on the importance of ATG proteins during MHV replication reached conflicting conclusions. Prentice et al. argued that components of the autophagy machinery are required for MHV replication while Zhao et al. demonstrated that autophagy was dispensable for the same process [65,66]. In particular, both teams monitored viral replication in murine embryonic fibroblasts (MEFs) knocked out for *ATG5*. The first group found that both MHV replication and DMV formation was impaired in *atg5^−/−^* knockout cells, while the second did not observe any effect on MHV life cycle in absence of *ATG5* [65,66]. The fact that MHV infection does not require intact ATG machinery was also later confirmed by another group using *atg7^−/−^* MEFs [67]. Data from both laboratories, however, established that the viral RTCs are co-localizing with endogenous LC3, which on one hand was in agreement with observations gained from SARS-CoV, but on the other hand was conflicting with results obtained with MHV by a third group [81,82]. These apparent discrepancies were subsequently explained by showing that endogenous LC3 but not ectopically expressed GFP-LC3 co-localizes with *CoV* RTCs [67].

Data from different groups strongly support an ER involvement in convoluted membranes and DMVs biogenesis, as those structures can be found connected to the ER and transmembrane nsp proteins can be glycosylated and localize to the ER when individually expressed [83,84]. The RTCs and DMVs, however, do not co-localize with marker proteins of the ER, ERGIC, and the Golgi [82,84] and disruption of the secretory pathway has no major effect on *CoV* replication [85]. This indicates that DMVs’ biogenesis might not involve the secretory pathway. In contrast, the ER-associated degradation (ERAD) tuning pathway, a vesicular transport route out of the ER, has been shown to be important for *CoV* infection [86]. ERAD allows for the degradation of misfolded ER proteins and it is negatively regulated during normal growing conditions, in absence of stress. This tuning down of the ERAD activity is mediated at least in part by small vesicles called EDEMosomes, which specifically capture key positive ERAD regulators such as EDEM1 and OS-9, and degrade them in compartments of the endolysosomal system [56]. Interestingly, EDEMosomes are decorated with non-lipidated from of LC3 (also called LC3-I) and their formation might require selected components of the ATG machinery, such as ATG5 [54,55]. Reggiori et al. eventually revealed that DMVs were associated with LC3-I and positive for both EDEM1 and OS-9, suggesting that MHV might actually highjack part of the membranes of the ERAD tuning pathway [67]. Although expression of a non-lipidable LC3 impaired DMVs biogenesis and viral replication, absence of EDEM1 and OS-9 had no effect. Thus, the authors hypothesized that one or more nsp proteins might associate with components of the machinery of EDEMosomes, such as a cargo receptor or a coat protein, to subvert these vesicles and generate the DMVs. LC3-I could be such a candidate but no interaction between LC3-I and MHV nsp proteins was detected using the yeast two-hybrid assay [67]. How MHV highjacks EDEMosomes and what the exact role of LC3-I is in this process are questions that remain unanswered.

Overall, *beta-CoV* interactions with autophagy and ATG proteins appear to be complex. Although MHV hijacking of LC3-positive EDEMosomes for its replication appears to be clearly established, this finding has not yet been extended to other *beta-CoV* or to other *CoV* in general. Co-localization between SARS-CoV RTCs and endogenous LC3, however, has been reported [81]. *Beta-CoV* do not require canonical autophagy for their life cycle [65,66,67] but it cannot be excluded a priori that they could need a non-canonical form of autophagy, independent from ATG5 and ATG7 [87]. Furthermore, while autophagy might be induced during infection or transient expression of single viral proteins [65,88], there is currently no evidence that this is directly regulated by *beta-CoV*. Indeed, autophagy stimulation could be part of a cellular response caused by either the presence of toxic exogenous proteins or ER stress induced by the massive production of viral proteins [89,90,91]. A recent study concluded that expression of nsp3 fragments derived from several *CoV*, which comprise the papain-like protease domain and one transmembrane segment, induce autophagy through direct binding to LC3 and BECLIN1 [92]. This conclusion, however, has to be carefully considered since the authors of this study observed GFP-LC3 puncta formation in cells ectopically expressing the nsp3 fragment but they did not examine whether these puncta are indeed autophagosomes. Moreover, the relevance of the use of a truncated form of nsp3 in absence of a *CoV* infection remains to be determined. Additional studies are thus needed to address the questions if and, eventually, how *beta-CoV* trigger autophagy.

### 3.4. Gammacoronavirus and Autophagy

*Gammacoronaviruses* (*gamma-CoVs*) are viruses that mainly infect poultry but are also transmissible to humans. They replicate in the respiratory tract and thus cause respiratory defects. The Infectious Bronchitis Virus, which causes major loses in the poultry industry, is the model virus for *gamma-CoV*. Similarly to other *CoV,* the IBV genome encodes several nsp proteins that help with replication and interfere with host cell functions.

Cottam et al. have reported that infections with IBV trigger the formation of endogenous LC3-positive puncta in host cells [88,93]. Interestingly, they noted that a fraction of these puncta partially co-localized with double stranded viral RNA. By screening several IBV nsp proteins, they found that ectopically expressed nsp6 localized with the ER and was able to autonomously induce the formation of GFP-LC3 puncta. This raised the question whether the GFP-LC3 puncta induced by nsp6 were EDEMosomes [67]. In contrast to EDEMosomes, however, formation of these GFP-LC3-positive vesicles required ATG5 and LC3 lipidation, suggesting that they are canonical autophagosomes [56,67,88]. Interestingly, nsp6 from MHV and SARS-CoV also induced GFP-LC3 puncta formation indicating that the nsp6-dependent mechanism for autophagy induction might be conserved among *CoVs*. Cottam et al. also argued that nsp6 might reduce the expansion of the autophagosomes as well, while maturation into autolysosomes is still possible [93]. These results have been obtained using ectopic expression of *nsp6* and thus the relevance of nsp6-mediated induction of autophagy during *CoV* infection remains to be explored.

## 4. Conclusions

The investigation of the potential interplay between *Nidovirales* and autophagy is still at its beginning. Nonetheless, it can already be firmly concluded that *Nidovirales* infections trigger autophagy in host cells. Several viral families and virus species such as *Torovirinae*, *Mesoviridae,* and *Roniviridae*, have yet to be investigated while for others, such as PRSSV and TGEV, opposite conclusions have been reached regarding whether autophagy induction is beneficial or detrimental for the viral life cycle. The apparent contradictory results could be due to the use of different cell lines and tissues, and/or virus strains. Some of these discrepancies could also be due to potential noncanonical functions of ATG proteins as was shown for MHV. Further investigations are therefore needed to reconcile these results. Another drawback of several of the studies cited in this review is the extensive use of drugs to modulate autophagy during viral infection. As none of the employed compounds inhibits autophagy specifically, they can have adverse effects on cellular or viral biology, making the interpretation of the results difficult. The genetic ablation of ATG proteins is a better option but it must be kept in mind that these factors are also part of other pathways [48,94]. As a result, it is crucial to compare the results obtained by depleting more than one ATG protein. Moreover, the few studies that have depleted ATG proteins have blocked the initial steps of the autophagic pathway (Figure 3 and Table 1) without analyzing the steps following the completion of an autophagosome. This is relevant since some viruses such as Influenza A or Epstein–Barr virus, have been shown to manipulate this part of the pathway and therefore it is critical to investigate whether it could also be the case for *Nidovirales* [95,96]. It also remains unclear which step of the virus life cycle is impacted, as most studies relied on assays quantifying general parameters such as the viral protein levels, the number of infected cells, and /or the number of produced infectious viral particles. Results that were obtained by studying viruses from other orders have revealed that autophagy and ATG proteins can practically play a key role in every step of a virus life cycle, from entry to assembly and egression [97].

While it is indisputable that large part of the investigated *Nidovirales* induces autophagy in host cells, it still remains unclear whether this is due to a subversion of autophagy by the virus or whether it is a physiological response to the cellular stress caused by either the infection or the transfection of single viral proteins. Future research should therefore also focus on the identification of a potential direct molecular link between viral and ATG proteins. Such studies could also pave the way to the development of novel antiviral therapies targeting the virus–autophagy interaction.

## Figures and Tables

**Table 1:** Summary of the current knowledge on autophagy contribution over the course of Arteriviruses and Coronaviruses infections.

Virus	Autophagy Role during Infection	Strategy Used to Modulate Autophagy	Infected Cell/Organ	References
TGEV	Antiviral	rapamycin, wortmannin, ATG5/ATG7/LC3 knockdown	Porcine ST cells	[54]
	Proviral	rapamycin, 3-MA, ATG5 knockdown	Porcine IPEC-J2 cells	[55]
PEDV	Proviral	rapamycin, 3-MA, ATG5/BECLIN1 knockdown	Simian Vero E6 cells	[56]
PRSSV	Proviral	rapamycin, 3-MA, BafA1, ATG7/BECLIN1 knockdown	Piglet thymus, simian Marc145 cells	[57,58,59,60,61,62,63,64]
MHV	None (LC3 unconventional use)	ATG5/ATG7 knockout, LC3 knockdown	MEFs, human HeLa and HEK293 cells	[65,66,67]
EAV	None (LC3 unconventional use)	ATG7 knockout, LC3 knockdown	MEFs, simian Vero E6 cells	[68]

Green shading indicates a pro-viral role for autophagy, grey shading indicates conflictual reports and blue shading highlights an unconventional role of autophagy-related (ATG) proteins. The table also provides the information about how autophagy has been experimentally manipulated in the studies addressing the role of this pathway with the indicated virus, the used cell types, and the references. 3-MA: 3-methyladenine; BafA_1_: bafilomycin A_1_, LC3: microtubule-associated protein light chain 3, MEFs: mouse embryonic fibroblasts.
